# Prenatal Diagnosis and Outcomes in Fetuses with Hemivertebra

**DOI:** 10.3390/genes13091623

**Published:** 2022-09-09

**Authors:** Hang Zhou, You Wang, Ruibin Huang, Fang Fu, Ru Li, Ken Cheng, Dan Wang, Qiuxia Yu, Yongling Zhang, Xiangyi Jing, Tingying Lei, Jin Han, Xin Yang, Dongzhi Li, Can Liao

**Affiliations:** 1 Department of Prenatal Diagnostic Center, Guangzhou Women and Children’s Medical Center, Guangzhou Medical University, Guangzhou 510620, China; 2 The First Clinical Medical College, Southern Medical University, Guangzhou 510515, China; 3 School of Medicine, South China University of Technology, Guangzhou 510641, China

**Keywords:** fetal hemivertebra, chromosomal microarray analysis, prenatal diagnosis, whole exome sequence

## Abstract

Background: There are few studies on the burden of chromosomal abnormalities and single gene disorders in fetal hemivertebra (HV). We aim to investigate the cytogenetic and monogenic risk and evaluate prenatal outcomes of fetal HV. Method: This study included fetuses diagnosed with HV divided into two groups: isolated HV and non-isolated HV. Data on other sonographic structural anomalies, chromosomal and sub-chromosomal abnormalities, monogenic variations detected by WES, and prenatal outcomes are recorded and reviewed. Results: Among 109 fetal HV cases, forty-seven (43.1%) non-isolated HV cases were associated with structural anomalies. Chromosomal test results were available in 58 cases, identifying six (10.3%) chromosomal aberrations involved in four isolated and two non-isolated HV. WES identified four (likely) pathogenic variants in three cases among 16 fetuses with HV, involving three novel variants, 1250G > T and c.1277G> inherited from parents, respectively, in DLL3 and c.7213C > A ** in the FLNB. The live birth rate (LB) was higher in the isolated fetal HV group than in the non-isolated group (67.7% (42/62) vs. 12.5% (12/47), *p* < 0.001). Conclusion: This study emphasizes the risk of cytogenetic abnormalities in isolated HV. WES yields a diagnostic rate of 18.3% in HV with normal CMA, probably aiding the prenatal counseling and management of fetal HV.

## 1. Introduction

Hemivertebra is the most frequent vertebral anomaly of congenital spinal abnormalities, such as congenital scoliosis and kyphosis, with an incidence of about 0.1–1.0% in live births [1]. The patients with HV deformity tend to experience spinal cord strain due to a loss of normal physiologic curvature of the spine. The HV may affect the development and function of the heart, lungs, and other vital organs by compression. Other associated structural malformations are observed in about 59.2–71.2% of fetuses with HV involving the cardiovascular system, genitourinary system, skeletal system, and central nervous system, etc. [1,2,3]. Moreover, 25.9% of children diagnosed prenatally with HV experience developmental delay [2]. Possible causes of fetal HV include failure of chondrification, spinal formation and development, and nutritional deficiency resulting from abnormal distribution of nutrients [4,5]. Despite this, few studies have been conducted on chromosomal abnormalities and single gene disorders in fetuses with prenatal HV.

As chromosomal microarrays (CMA) and whole exome sequence (WES) have been rapidly developed and applied to prenatal diagnosis, CMA improves the diagnostic rate by 6–10% [6,7,8], and WES yields an additional diagnostic rate of approximately 10% in fetal sonographic structural abnormalities, respectively [9]. Although a few small-sample studies suggest that chromosomal abnormalities are unlikely in isolated fetal HV, there is still debate in clinical practice about how to use and apply cytogenetic tools for fetal HV due to the lack of evidence. In a systematic review, 326 fetal HV were found to have 5% cytogenetic abnormalities, including partial tetrasomy 4q, mosaic trisomy 4, mosaic trisomy 7, mosaic trisomy 9, mosaic trisomy 18q, mosaic trisomy 18; trisomy 7, trisomy 15q with monosomy 6q, partial trisomy 22; duplication of 2p; 4p- deletion, 17p deletion, 18p deletion, 18q22.2 deletion, 22q13.3 deletion; ring chromosome 21, and Fanconi’s anemia [10]. Furthermore, HV is linked with numerous genetic disorders, including *DLL3*, *MESP2*, *LFNG, HES7*, *TBX6*, *RIPPLY2*, *GDF6,* and *EBP* genes, etc. [11]. However, rather than exome sequencing, most of the studies on the genetic causes of HV are typically conducted using gene panels or particular gene detection techniques. WES is still understudied and underutilized when it comes to fetal HV, causing confusion and challenges in genetic counseling and management for pregnancies with fetal HV in the prenatal setting.

This study investigates chromosomal and sub-chromosomal abnormalities as well as monogenic conditions in fetal HV and assesses the efficacy and performance of both CMA and WES in pregnancy. In addition, the perinatal and postnatal prognosis of fetal HV is evaluated.

## 2. Materials and Methods

A retrospective study was conducted to identify the cases with fetal hemivertebra identified by prenatal ultrasound or nuclear magnetic resonance imaging (MRI) from 2015 to 2021 in the Guangzhou Women and Children’s Medical Center. This study was approved by the Ethics Committee of the Guangzhou Women and Children’s Medical Center. The HV is diagnosed if the strong echoes of triangular and irregular solitary bony structures are found within the vertebral columns, and the two strong echo lines of the spine are not parallel. Parents were offered chromosomal microarray analysis (CMA) after completing pretest counseling and obtaining informed consent. Demographic and genetic data were collected, including maternal age, gestational age at which HV suspicion arose, stage of the disease, other structural abnormalities associated with HV, and chromosomal and genetic test results of the invasive procedures and perinatal outcomes, etc. The acronym VACTERL is defined by the presence of at least three of the following: Vertebral, Anal, Cardiac, Tracheoesophageal fistula or Esophageal atresia, Renal, and Limb anywhere. The information on postnatal outcomes was obtained from the medical record system and telephone follow-up. Preterm birth was defined as a gestational age < 37 weeks after gestational age was assessed by maternal menstrual history and ultrasound in the first trimester. The pediatric medical records were reviewed to investigate the onset of scoliosis or other spinal abnormalities and whether an operation was needed.

The DNA sample was extracted by chorionic villus cells, amniocytes, umbilical cord blood cells, and peripheral blood lymphocytes by using a QIAamp DNA Blood Midi/Mini kit (QIAGEN GmbH; Hilden, Germany). The quantitative fluorescent polymerase chain reaction (QF-PCR) was used to exclude maternal cell contamination and abnormalities of chromosomes 21, 18, 13, and sex chromosomes. The samples were subjected to CMA when there were normal QF-PCR results. CMA was conducted by using Affymetrix CytoScan HD/750K array with a single-nucleotide polymorphism array (SNP array) and array-based comparative genomic hybridization (aCGH) platforms at resolutions of 10 and 100 kb, respectively, according to the manufacturer’s protocol (Affymetrix Inc., Santa Clara, CA, USA) as in our previous study [12]. The genome built was referred to as GRCh37/hg19. The classification of the copy number variants was according to joint consensus recommendations of the American College of Medical Genetics and Genomics and ClinGen [13]. The pathogenic CNVs, likely pathogenic CNVs, and variants of unknown significance (VUS) are recorded and documented, but likely benign and benign VUS are not considered. Parental CMA is advised for couples to determine the source of CNVs when fetal CNVs are discovered. The samples were subjected to Trio-WES once there were normal QF-PCR and CMA results. According to the manufacturer’s protocol, the DNA samples were enriched using Agilent SureSelect human exome capture probes (V6, Life Technologies, Carlsbad, CA, USA). The pair-end 150-bp reads’ libraries were sequenced using Hiseq XTen (Illumina, Inc., San Diego, CA, USA). NextGENe v2.4.1.2 software (SoftGenetics, State College, PA, USA) was used for variants calling. After filtering out the synonymous and common SNPs (MAF > 0.1%), rare variants with high confidence were considered disease-causing candidates. Variant annotation was further confirmed through literature and population databases, including 1000 Genomes, dbSNP, GnomAD, Clinvar, HGMD, and OMIM. Multiple computational algorithms, including SIFT, MutationTaster, PolyPhen2, PROVEAN, CADD, Human Splicing Finder, MaxEntScan, and NNSplice, were used to evaluate the pathogenicity of the candidate gene variants. The interpretation of the variants was performed according to the American College of Medical Genetics’ guidelines [14].

Statistical analysis was performed using the IBM statistical program SPSS 25.0. The mean and range were used to describe the demographical characteristics. The Chi-square test was used for categorical data in appropriate cases. A *p*-value < 0.05 was considered statistically significant.

## 3. Results

**Figure 1:**
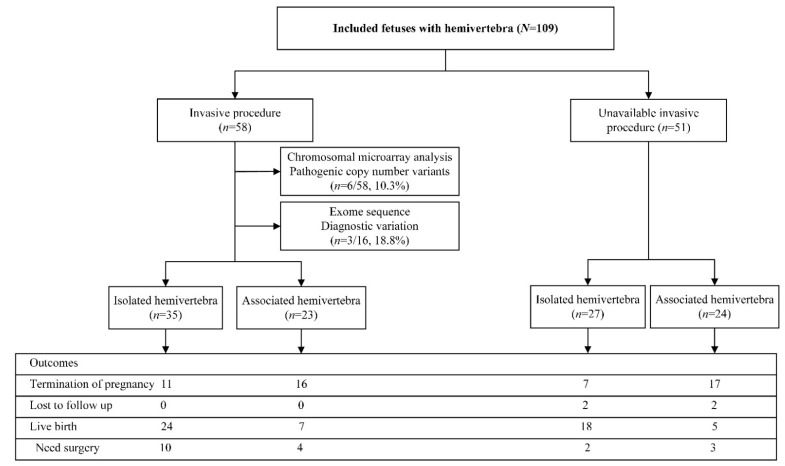
Flowchart of this study.

A total of 109 cases with prenatal HV were included in the study flowchart shown in Figure 1. There were 58 fetuses with chromosomal test results from invasive procedures with six (10.3%) diagnostic pathogenic copy number variants. The whole exome sequence yielded a detection rate of 18.8% in 16 fetuses with HV. The outcome included 51 terminations of pregnancy, four lost follow-ups, and 54 live births, including 42 full-term births and 12 preterm births. Nine (33.3%) of the 27 terminations of pregnancies with the available results of the invasive procedures used the prenatal genetic diagnosis information to assist them in making the choice to terminate the pregnancy, seven cases due to the diagnosis of other malformations, and the voluntary abortion in the remaining 11 cases. The live birth rate (LB) was higher in the isolated fetal HV group than in the non-isolated group (67.7% (42/62) vs. 12.5% (12/47), *p* < 0.001). Preterm births occurred in 19.4% (8/42) of isolated HV group and 33.3% (4/12) of non-isolated group (*p* = 0.431). Among the live birth cases, there was an operation rate of 28.6% (12/42) in the isolated group and 58.3% (7/12) in the non-isolated group (*p* = 0.087). The pathogenic or likely pathogenic variants identified on CMA or WES results only, and the clinical outcomes of fetal HV are presented in Table 1. The details of genetic and clinical outcomes of fetal HV are presented in Table 1. Spinal surgery was required in 19 cases. The mean follow-up period was 2.3 years (range 0.2–8.1 years). All of the live birth cases were found to be without developmental delay except that one female case with HV combined with a butterfly vertebra, who had normal CMA, and later developed seizures postnatally. 

The mean maternal age was 30.7 years (range 19.0–42.0 years), and the mean gestational age at suspected HV was 23.9 weeks (range 15^+1^–32^+0^ weeks). Among 62 (56.9%) cases with isolated HV, 44 fetuses had a single HV at the cervical (*n* = 3), thoracic (*n* = 24), lumbar (*n* = 16), and sacral (*n* = 1) vertebras. Eighteen fetuses were found with multiple stages of vertebral involvement. Another 47 (43.1%) non-isolated HV cases were associated with structural anomalies. Table 2 shows the details of associated anomalies identified sonographically in fetuses with HV. The skeletal system (19.1%) and genitourinary system anomalies (19.1%) were the most frequently associated structural anomalies, followed by the cardiovascular system (16.2%), central nervous system (10.3%), craniofacial abnormalities (8.8%) and gastrointestinal system (5.9%). Other structural anomalies included polyhydramnios, fetal growth restriction, oligohydramnios, and pleural effusion. In addition, VACTERL syndrome was identified in three cases.

CMA detected six (10.3%) clinically significant variants (pCNVs) and five (8.6%) variants of uncertain significance (VOUS). The clinically significant variants included mosaic trisomy 21, maternal uniparental disomy for chromosome 15, 16p11.2 microdeletion, 17p11.2 microdeletion, 8q24.3 microduplication, and 21q22.2q22.3 microdeletion involved in four fetuses with isolated HV and two with non-isolated HV (Table 3). Parental CMA results were available in five cases with P/LP or VUS, but the remaining patients refused the test because it costs more than USD1000. Among the five cases, the CNVs identified by CMA were de novo in patients 1, 2, 3, and 6. However, the VUS detected in patient 7 was inherited from his father who did not exhibit associated phenotypes. The 32 cases with negative CMA did not undergo WES mainly because the patients rejected trio-WES due to its high cost, which can reach USD1500 in China.

As shown in Table 4, the whole exome sequence identified four (likely) pathogenic variants in three genes and three variants of uncertain significance (VUS) in normal CMA cases. The pregnancy of patient 12 with a two-times birth history of spinal anomalies, identified with multiple stages of HV and multi-segment fusion, was detected with two novel and missense mutations, NM_203486.3, c.1250G > T (p. Cys417Phe) and c.1277G > A (p. Cys426Tyr) inherited from the father and mother, respectively, in the *DLL3* gene, with autosomal recessive inheritance mode, leading to spondylocostal dysostosis 1 (OMIM: 277300). In patient 13, the fetus was found with thoracic HV and short limb long bone (<−3SD) and identified with a de novo heterozygous variant in the *EBP* gene, NM_006579.2, c.328C > T (p. Arg110Ter) with AD mode, which could result in X-linked dominant chondrodysplasia punctata and MEND syndrome (OMIM: 302960, 300960). Patient 14 was diagnosed with thoracic HV associated with sacrococcygeal spina bifida. In this fetus, WES detected a mutation in the *FLNB* gene, NM_001164317.1, c.7213C > A ** (p. Arg2405Ser). This variant was de novo, pathogenic, and nonsense and associated with Larsen syndrome (OMIM: 150250).

## 4. Discussion

Hemivertebra is the most common cause of congenital vertebral abnormalities, with an approximate incidence of 0.1–1.0% of births [1]. With the wide application and use of chromosomal microarray analysis and whole exome sequence in the prenatal setting, chromosomal, sub-chromosomal, and genetic etiologies of prenatal skeletal abnormalities have been well studied and understood [6,15]. However, little research has been conducted on the cytogenetic and monogenic burden and outcomes in fetuses with HV. The study aimed to evaluate the efficacy of CMA and WES, and the perinatal and postnatal outcomes in fetal HV.

There was a high prevalence of associated structural anomalies in prenatal fetuses with hemivertebra. Previous studies found co-existing abnormalities in 59.2–71.2% of fetal HV cases [1,2,3]. This study found 43.1% of cases diagnosed with other structural defects, lower than the previous data. As a tertiary referral center, there was likely a selective referral bias, and more minute and minor changes in fetal vertebral columns could be identified by the increasingly better resolution of the ultrasound and magnetic resonance machine. Furthermore, similar to previous studies [4,11], in these fetuses with associated anomalies, the incidence was high in the genitourinary, skeletal, and cardiovascular system, followed by central nervous systems, etc. Additionally, it was important to note that there were three HV fetuses with fetal growth restriction. A recent study proved the association between the supernumerary hemivertebra and intrauterine growth restriction [16]. Thus, this suggested that it was necessary to conduct a thorough anomaly scan, biometry monitoring and echocardiogram for fetal HV.

It seems that chromosomal abnormalities are not usually associated with isolated fetal HV [1,2,16]. In addition, a recent systematic review of prenatal cytogenetics of the fetal hemivertebra showed that cytogenetic abnormalities accounted for 5% of 246 fetal HV cases, and what is noteworthy is that all of the cases had prenatally (29.0%) or postnatally (71.0%) associated anomalies [10]. However, isolated fetal HV was indeed associated with chromosomal and sub-chromosomal abnormalities. In our series, it was noteworthy that there were four fetuses with isolated HV and two with non-isolated HV among all six with cytogenetic diagnoses. It implied that chromosomal abnormalities could be present in fetuses with isolated HV. Because some co-existing anomalies may have been missed by prenatal ultrasound and likely observed postnatally, the so-called “prenatal isolated HV” may not actually represent true isolated HV. In short, a diagnostic yield of 10.3% warrants the application of CMA in pregnancies with HV. 

Congenital spinal abnormalities can result from the 16p11.2 microdeletion syndrome [17]. We found two cases of 16p11.2 microdeletion syndrome in patients 5 and 6 with isolated HV. According to a postnatal study of congenital scoliosis, 10% of affected cases could be explained by the 16p11.2 microdeletion [18]. Additionally, a study of prenatal sonographic features in 16p11.2 microdeletion syndrome found that 41.7% of fetuses had spinal defects, including HV and butterfly vertebra [19]. The TBX6 gene, which is located on the 16p11.2 chromosome, has been shown through animal studies to be crucial in congenital spinal abnormalities. The primary mechanism involves the haploinsufficiency mechanism, which leads to reduced regulation of downstream genes due to mislocalization of the T-Box transcription factor 6, thereby dysregulating the Notch signaling pathway, which is essential for somite development [20,21,22,23]. However, it is crucial to note that increased doses of TBX6 are responsible for congenital malformations of cervical vertebrae [24]. This suggests that precise dosages of the TBX6 gene have a crucial impact on spinal development. Furthermore, the hemivertebra and scoliosis resulted from the nonfunctional allele in combination with another common hypomorphic allele haplotype T-C-A (three common single nucleotide polymorphisms: rs2289292, rs3809624, rs3809627) in *TBX6* [25]. The frequency of the T-C-A haplotype reaches 44% among Han Chinese individuals in the 1000 Genomes Project [26]. Therefore, the allelic haplotype T-C-A of *TBX6* should not be overlooked in hemivertebra and congenital scoliosis. 

Previous research indicated that the whole exome sequence could provide approximately 8.5–10.0% diagnostic yield in fetal structural anomalies detected by ultrasound in normal CMA [9,27]. While the application and utility of WES in fetal HV was still unclear, our data showed that WES improved an 18.8% genetic diagnostic rate for fetal HV over CMA. All of the three cases with diagnostic variants were detected with multiple HV, and two of them had short limb bone and spinal bifida, respectively. Furthermore, the whole exome sequence can identify additional novel variants and findings. This was the first report of the association between fetal multiple HV and *DLL3*, demonstrating the prenatal phenotype for *DLL3*. Two novel variants in the *DLL3* gene, transmitted from the parents, were detected in patient 12 with multiple HV, c.1250G > T (p. Cys417Phe), and c.1277G > A (p. Cys426Tyr). In addition, it suggested that each sibling of an affected individual had a 25% chance of being affected by the two novel variants of the *DLL3* gene in this family. In the genetic counseling of this couple, there were at least two options. First, the chorionic villus sampling is performed to exclude the two variants of the *DLL3* gene in a subsequent pregnancy as early as in the first trimester. Secondly, pre-implantation for monogenic disease (PGT-M) can be a selection to rule out the two variants of the *DLL3* gene during the in vivo fertilization (IVF) process. Additionally, a novel mutation in the *FLNB* gene, c.7213C > A ** (p. Arg2405Ser), was identified in patient 14. This variant was de novo and absent from the parents, meaning that the recurrent risk of this variant would be no more than 3% in the following pregnancy, except for the parental germline mosaicism. Therefore, if fetuses with HV have been identified by ultrasound or MRI, particularly when multiple segmentation of HV or co-existing structural abnormalities have been diagnosed, whole exome sequencing could be offered to provide information on genetic variants for cases with normal CMA. 

The isolated HV group had better prenatal and postnatal outcomes than the non-isolated group. Our data showed higher birth rates in isolated prenatal cases with hemivertebra. Similar to previous investigations, fetal survival can be lowered if additional structural defects are found [3]. Additionally, preterm rates and surgery rates appeared to be lower in isolated HV cases compared to hemivertebra, despite there being no significant difference. Surprisingly, 53 live births achieved good results with a mean follow-up of 2.3 years, and no developmental delay was found except in one patient with epilepsy. Long-term follow-up care is required. Prenatal CMA was normal in 31 out of the 54 birth cases, including 13 cases that were undiagnosed through WES. It is intriguing to see if patients with normal cytogenetic testing and those without prenatal genetic testing have different long-term follow-up prognosis. To sum up, our study supported the favorable outcome in fetuses with isolated HV diagnosed with normal CMA or WES.

There are several limitations in this study. The recall bias may be present in this retrospective study. Prenatal skeletal abnormalities can be associated with methylation abnormalities and deep intron variations not detectable by CMA or WES. This is a small-sample study, and more research and evidence are needed to investigate the genetic burden of fetal HV.

## 5. Conclusions

In conclusion, we confirmed the high prevalence of co-existing anomalies in fetuses with hemivertebra. This study showed that the risk of chromosomal and sub-chromosomal abnormalities identified by CMA was as high as 10.3% for fetal hemivertebra with and without other structural defects. WES can yield an 18.8% diagnostic rate in fetuses with normal CMA for fetal hemivertebra, particularly when multiple vertebras are affected and accompanied by other structural abnormalities. Achieving prenatal chromosomal and genetic diagnosis by CMA and WES in fetal hemivertebra provides further information for management and couple counselling.

## Figures and Tables

**Table 1:** Genetic and clinical outcome in fetuses identified sonographically in fetuses with hemivertebra.

Characteristics	Isolated HV (%, *n*/*N*)	Non-Isolated HV (%, *n*/*N*)	*p*-Value
Diagnostic rate of CMA	11.4, 4/35	8.7, 2/23	1.000
Diagnostic rate of WES	11.1, 1/9	28.6, 2/7	0.550
Live birth rate	67.7, 42/62	12.5, 12/47	<0.001
Operation rate	28.6, 12/42	58.3, 7/12	0.087

CMA, chromosomal microarray analysis; WES, whole exome sequence; HV, hemivertebra.

**Table 2:** Associated anomalies identified sonographically in fetuses with hemivertebra.

Associated Anomalies	Number
**Skeletal system**	**13 (19.1%)**
Butterfly vertebra	5
Talipes equinovarus	2
Short long bone	2
Ectrodactyly	1
Congenital club hand	1
Partial absence of 7th ribs	1
Preaxial polydactyly	1
**Genitourinary system**	**13 (19.1%)**
Renal agenesis or hypoplasia	5
Polycystic kidney dysplasia	2
Unilateral hydronephrosis with or without ureter ectasis	4
Hypospadias	1
Renal duplication	1
**Cardiovascular system**	**11 (16.2%)**
Ventricular septal defect	3
Hypoplastic heart	1
Pulmonary atresia	1
Tetralogy of Fallot	1
Aortic stenosis	1
Coarctation of aorta	1
Cardiac Malposition	1
Complete atrioventricular septal defect	1
Complete transposition of great artery	1
**Central nervous system**	**7 (10.3%)**
Spina bifida with or without meningocele	5
Microcephaly	2
**Craniofacial**	**6 (8.8%)**
Cleft lip and/or palate	5
Anterior nasal excrescence	1
**Gastrointestinal system**	**4 (5.9%)**
Small stomach	2
Congenital megacolon	1
Esophageal atresia with tracheoesophageal fissure	1
**Others**	**14 (20.6%)**
Polyhydramnios	8
Fetal growth restriction	3
Oligohydramnios	2
Pleural effusion	1

**Table 3:** Pathogenic copy number variation and VUS identified by CMA in prenatal hemivertebra cases.

Patient	MA	GA at the Suspicion of Hemivertebra	Affected Vertebra	Associated Anomaly	Microarray Result	Length	Type	Classification
1	31.0	25 + 6	L1	Pulmonary atresia	arr [hg19] 8q24.3 (140131302_ 146295771) X3 arr [hg19] 21q22.2q22.3 (39737188_ 48093361) X1	6.16 Mb 8.36 Mb	Duplication Deletion	P P
2	29.0	21 + 1	T10, T12, L2, L3	/	arr [hg19] (15)X2 hmz	/	UPD 15	P
3	33.0	23 + 2	L3, S1, S2	Renal dysplasia, ventricular septal defect, pulmonary atresia, right aortic arch, persistent left superior vena cava, and sacrococcygeal dysplasia	arr [hg19] (21) X2~3	33.08 Mb	Mosaic	P
4	33.5	26 + 1	T11	/	arr [hg19] 17p11.2 (16657319_20417235) X1	3.76 Mb	Deletion	P
5	30.0	22 + 3	T11	/	arr [hg19] 16p11.2 (29428531_30190029) X1	761 Kb	Deletion	P
6	23.0	24 + 1	L1	/	arr [hg19] 16p11.2 (29567295_30240227) X1	611 kb	Deletion	P
7	25.0	25 + 0	T6	/	arr [hg19] 15q13.2q13.3 (31104221_32915723) X3	1.81 Mb	Duplication	VUS
8	29.0	25 + 0	C7	Spina bifida, renal agenesis	arr [hg19] 6p22.3 (21730212-21957713) X1	228 Kb	Deletion	VUS
9	28.0	27 + 2	L3	left renal hypoplasia, right hydronephrosis, oligohydramnios	arr [hg19] 2q37.3 (238143761_238617753) X3	474 Kb	Duplication	VUS
10	31.4	26 + 3	T3, T8	/	arr [hg19] 5q31.1 (135273369_135477266) X3	204 Kb	Duplication	VUS
11	24.2	27 + 5	L5	/	arr [hg19] 6q23.3q24.1 (138266085_139126324) X3	806 Kb	Duplication	VUS

MA, maternal age; GA, gestational age; P, pathogenic; VUS, variation of uncertain significance; UPD, Uniparental diploid; C, cervical vertebra; T, thoracic vertebra; L, lumbar vertebra; S, sacral vertebra.

**Table 4:** Diagnostic variants detected by whole exome sequence in fetuses with hemivertebra.

Patient	Ultrasound Findings	Gene	Transcripts	Variant	Origin	Inheritance	Classification	Zygosity	Condition
12	Multiple HV	DLL3	NM_203486.3	c.1250G > T (p. Cys417Phe)	Pat	AR	LP	Het	Chondrodysplasia punctata, X-linked dominant
				c.1277G > A (p. Cys426Tyr)	Mat	AR	LP	Het	
13	Multiple HV, short long bone	EBP	NM_006579.2	c.328C > T (p. Arg110Ter)	De novo	AD	P	Het	Spondylocostal dysostosis 1, autosomal recessive
14	Multiple HV, spinal bifida	FLNB	NM_001164317.1	c.7213C > A ** (p. Arg2405Ser)	De novo	AD	LP	Het	Larsen syndrome
15	L1 HV	PTCH1	NM_000264.3	c.2687C > T p. (Pro896Leu)	Mat	AD	VUS	Het	Basal cell nevus syndrome
16	L3 HV	ERCC6	NM_000124.3	c.3061A > G p. (Ile1021Val)	Pat	AR	VUS	Het	Cockayne syndrome B
17	L1 HV	RBM10	NM_001204468.1	c.1980 + 7G > C	Mat	XR	VUS	Hemi	TARP syndrome

HV, hemivertebra; Pat, Paternal inherited; Mat, Maternal inherited; Het, heterozygous; AR, autosomal recessive; AD, autosomal dominant; LP, likely pathogenic; P, pathogenic; VUS, variant of uncertain clinical significance; XR, X-linked recessive; Hemi, hemizygous.

## Data Availability

The original contributions presented in the study are included in the article, further inquiries can be directed to the corresponding author.
